# Extraction of Rubidium and Cesium from a Variety of Resources: A Review

**DOI:** 10.3390/ma18143378

**Published:** 2025-07-18

**Authors:** Heyue Niu, Mingming Yu, Yusufujiang Mubula, Ling Zeng, Kun Xu, Zhehan Zhu, Guichun He

**Affiliations:** College of Resources and Environmental Engineering, Jiangxi University of Science and Technology, Ganzhou 341000, China

**Keywords:** rubidium, cesium, silicate minerals, pegmatite minerals, salt lake brine

## Abstract

In recent years, with the development of science and technology and the transformation of economic structures, rubidium and cesium have gradually become indispensable rare metal resources as important materials for high-tech industries. However, the relationship between supply and demand of resources is unbalanced, industrial demand is much higher than production, and the rubidium and cesium resources in hard rock minerals such as traditional pegmatite minerals are no longer enough to support global scientific and technological upgrading. There is therefore an urgent need to expand sources of resource extraction and recovery to meet market demand. This paper summarizes the current feasible technologies for extracting rubidium and cesium from pegmatite minerals, silicate minerals, salt lake brines and other potential resources.

## 1. Introduction

As rare metal resources, rubidium and cesium, with their outstanding photoelectric properties and excellent thermal and electrical conductivity, offer broad application prospects and development potential in traditional industrial sectors such as electronic devices, catalysts, and specialty glasses [1,2,3]. With the incessant advancement of global science and technology, developed countries are increasing their investment in research and development in the field of new energy conversion technology and emerging communication technology, while developing countries are showing a significant growth trends in the demand for high-quality development in high-tech fields such as national defense industries, aerospace, electronic information and biomedicine [4,5,6,7]. Due to their unique physical and chemical properties, the application scope of rubidium and cesium is continuously expanding. Their core status in fields such as new energy development is becoming increasingly prominent, making them key strategic resources that underpin the development of modern high-tech industries.

Rubidium and cesium exhibit unique advantages in numerous fields due to their extremely high ionization tendencies and strong absorption properties, as shown in Table 1. In the field of new energy, taking advantage of the highly ionizable characteristics of rubidium and cesium, the use of rubidium-containing materials in magnetohydrodynamic generators can significantly enhance the efficiency of converting thermal energy into electrical energy. It can improve the thermal conversion efficiency of nuclear power plants by approximately 32%. In the aerospace field, cesium ion propulsion systems enable high-speed cruising at a speed of 1.6 × 10^5^ km·h^−1^, and the addition of trace amounts of rubidium significantly enhances the stability of plasma. In trajectory measurement and calculation of spacecraft, in the oscillator of a cesium atomic clock a gram-level amount of cesium material enables frequency stability to reach the order of 1 × 10^−14^, which is three orders of magnitude higher than that of a quartz oscillator [5,6,7]. Rubidium and cesium have extensive applications and high adaptability, and there is a shortage of supply in the market to meet the demand.

**Figure 1:**
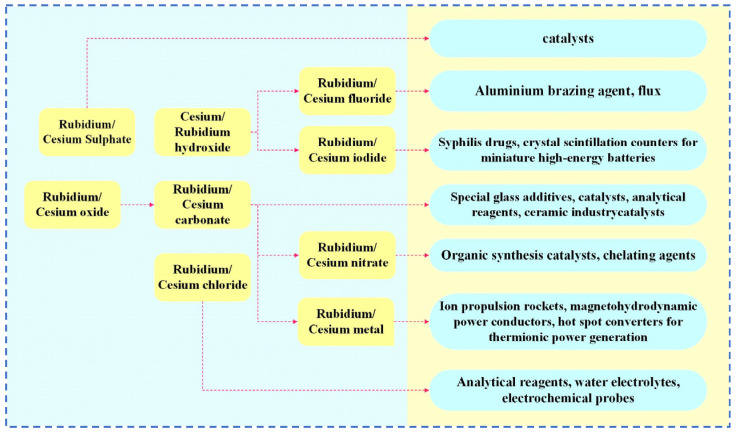
Rubidium and cesium products in the industry.

**Figure 2:**
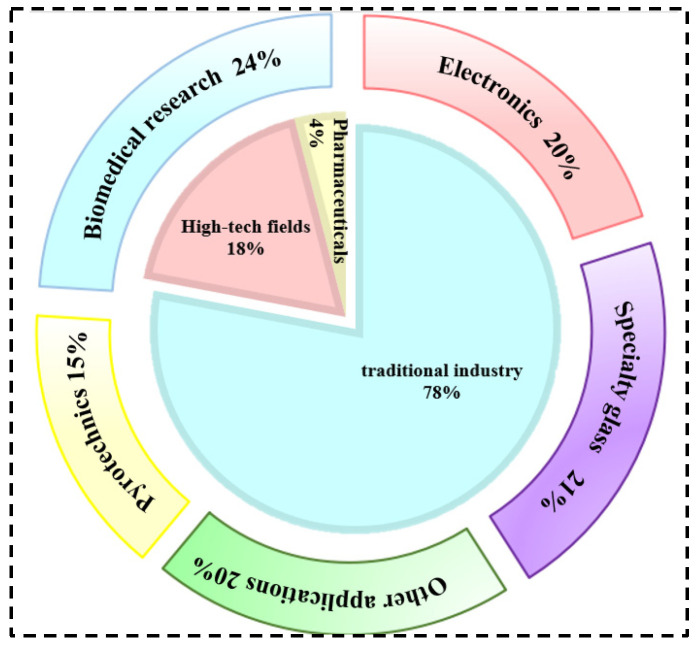
Rubidium and cesium traditional industry application areas and global industry structure.

Rubidium and cesium are elements in the same main group. Their physical and chemical properties and atomic radii are similar. Therefore, in many industrial applications, rubidium and cesium can be substituted for each other. However, rubidium often occurs associated with lithium and cesium deposits, and there are few independent deposits. It is more difficult to obtain than cesium [1,3,8]. Therefore, cesium has a wider range of applications. Currently, rubidium and cesium products in the market are mainly supplied in the form of rubidium/cesium salts. Among them, (Rb)_2_CO_3_, (Cs)_2_CO_3_ [9] as well as CsOH and RbOH are the most widely used in the industry, as shown in Figure 1. About 78 per cent of rubidium and cesium resources in the global market are used in the preparation of traditional products such as electronics, special glass and biomedical products. With the continuous advancement of scientific and technological innovation, the applications of rubidium and cesium in high-tech products such as atomic clocks, magnetohydrodynamic generators and ion thrusters have gradually expanded. They already account for 18% of the total and the demand is increasing year by year, as shown in Figure 2. Rubidium and cesium are transitioning from traditional industrial raw materials to strategic resources. With the continuous expansion of the rubidium and cesium market, they have gradually become high-consumption resources.

**Figure 3:**
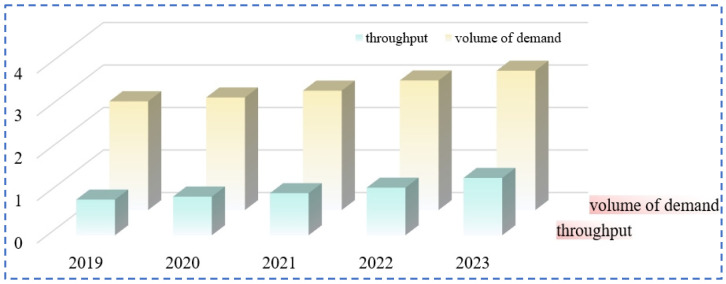
Rubidium and cesium market demand and rubidium and cesium supply.

Rubidium and cesium are among the alkali metals with the lowest contents in the earth’s crust. Rubidium has a content of 0.028% in the Earth’s crust, specifically ranging from 5.1 × 10^−5^% to 3.1 × 10^−4^%, and it ranks 16th in terms of elemental abundance. The content of cesium in the Earth’s crust is 1.2 × 10^−8^% to 1 × 10^−5^%, ranking 40th in terms of elemental abundance. The main forms of global rubidium and cesium resources include pegmatite-type, granite-type, salt lake brine-type and brine geothermal spring-type [5,10,11] (as shown in Table 2). They usually coexist with elements such as lithium and potassium. The minerals with industrial development value mainly include lepidolite, pollucite, orthoclase, etc. (as shown in Table 3). About 80% of the rubidium and cesium resources in salt lake brines have not been effectively developed. With the rapid transformation of the global economic structure, the market demand for rubidium and cesium shows a continuous growth trend (Figure 3). However, restricted by the existing extraction technologies, there is already a significant gap in the global supply of rubidium and cesium resources at present.

**Figure 4:**
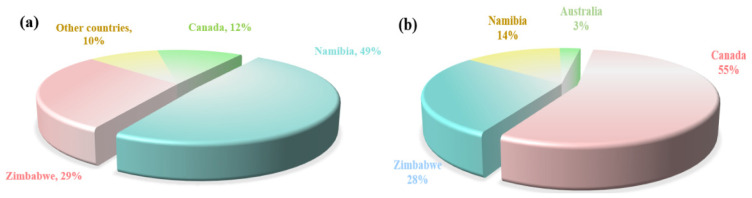
Global share of rubidium-(**a**) and cesium-proved geological reserves (excluding China) (USGS) (**b**).

The globally extractable rubidium and cesium resources are highly concentrated. According to data released by the United States Geological Survey, the proven reserves of rubidium oxide and cesium oxide globally (excluding China) are 102,000 tons and 220,000 tons, respectively. These resources are mainly distributed in four countries: Canada, Zimbabwe, Namibia and Australia. The specific distribution is shown in Figure 4. China is rich in rubidium and cesium resources, with its reserves ranking among the highest in the world. These resources are mainly stored in Jiangxi, Hunan, Xinjiang, Qinghai and other places. In places such as Qinghai and Tibet, rubidium and cesium are mainly distributed in the form of brine in salt lakes and geothermal water. However, due to their low content, they mainly exist in the form of chemical compounds and coexist with elements in the same main group that have similar chemical properties (such as lithium, sodium, etc.). Therefore, they currently do not have the value of independent development and utilization. In places such as Jiangxi, rubidium and cesium mainly exist in the form of rubidium oxide and cesium oxide in extractable solid ores. Cumulative hard rock cesium resources in China’s major mining areas hold 68,000 tonnes and 55% of rubidium resources are in Jiangxi pegmatite ores, the vast majority of which are endowed in lithium ores in the form associated [6,12]. Limited by technology, in the industrial field rubidium and cesium are often treated as by-products of lithium ores, which causes a certain amount of waste of rubidium and cesium resources. Therefore, there is an urgent need to develop new extraction technologies for rubidium and cesium to increase their production, meet market demands, and support the global transformation of science and technology.

With the continuous growth in the global industrial demand for rubidium and cesium resources, their consumption has increased significantly, resulting in the growing scarcity of these two resources, which makes it difficult to support the development of emerging industries. At present, in industrial production, the sources for extracting rubidium and cesium resources are relatively sparse, and the existing technologies have limitations. The resource recovery rate fails to meet the market demand for rubidium and cesium. Therefore, it is necessary to explore diversified ways of obtaining resources to alleviate the problems such as the constraints on industrial development caused by the shortage of rubidium and cesium resources.

Currently, rubidium and cesium resources are often extracted and recovered from pegmatites such as lepidolite, carbonate minerals such as pollucite and salt lake brines, and the leaching solutions of associated mica ores. However, rubidium and cesium resources can also be found in other potential resources, such as flotation tailings and geothermal water. If the full recovery of rubidium and cesium from these resources can be achieved, it will not only greatly alleviate the global shortage of rubidium and cesium resources but also reduce resource waste.

## 2. Minerals of the Mica Group

**Figure 5:**
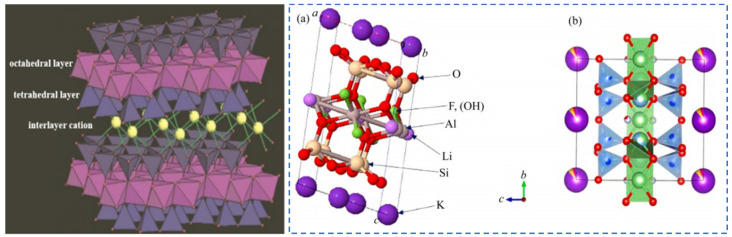
Mica crystal structure [13], schematic diagram of leucite T-O-T structure (**a**) [14], and (**b**) crystallography 365, 2014. https://www.iycr2014.org/learn/crystallography365/articles/20140912, accessed on 11 November 2024.

Minerals of the mica group have a unique structural characteristic of special layered silicate minerals. The crystal structure consists of silicon–oxygen tetrahedral sheets (T) and octahedral sheets (O) that are alternately connected in a T-O-T pattern to form structural unit layers. Alkali metal cations such as K, Na and Cs fill between the layers, as shown in Figure 5. During the mineralization process of minerals in the mica group, isomorphic substitution can occur. Rare elements Rb and Cs, which are also alkali metals, can substitute for K. Moreover, rare metals such as Li and Nb can partially replace elements like Al, Fe and Mg at the octahedral positions.

Rubidium and cesium are known as “rare and dispersed elements”. In nature, except for pollucite, they rarely exist as independent minerals and mostly exist as trace elements in other minerals. Rubidium and cesium have similar chemical properties to potassium and sodium. Therefore, Rb and Cs can easily displace K in the interlayer positions of mica through isomorphic substitution, becoming common trace element minerals in mica [13], and they are enriched at the edges of mica sheets or around the cleavage seams.

According to the classification principles of mica announced by the Mineral Nomenclature Commission of the International Mineralogical Association, lepidolite belongs to the lithium-bearing light-colored mica of the mica series composed of polylithionite and zinnwaldite, while annite-lithian belongs to the lithium-containing dark-colored mica of the mica series consisting of iron leaf mica-lithium polysilicon mica. During the mineralization process of lepidolite, Li undergoes isomorphic substitution reactions and, at the same time, the associated rare elements substitute for K, resulting in the chemical composition exhibiting diversity [13,15,16]. According to the differences in the elemental contents of lepidolite, annite-lithian, which has properties similar to those of lepidolite, evolves during the mineralization process. It exists as a transitional mineral and will eventually be transformed into lepidolite ore.

### 2.1. Lepidolite

Lepidolite is one of the minerals in the mica family and generally only exists in granite pegmatites. The chemical formula of lepidolite is K{Li_2−_xAl_1+x_[Al_2x_Si_4−2x_O_10_](F,OH)_2_} (x: 0~0.5), and its ideal chemical formula is KLi_1.5_Al_1.5_[AlSi_3_O_10_]F_2_. As a carrier mineral for rare metals [17], lepidolite hosts a variety of associated rare metal resources (such as rubidium, cesium, potassium, etc.) which exist in the form of oxides. In addition, lepidolite also contains various elements such as Al, Fe, Si, Mg and 2–4% of F, etc., which are mainly present in the lattice of its aluminosilicate. During the ore-forming process, Li will undergo isomorphic substitution reactions with Mg, Fe, Al, etc., and K will be replaced by associated rare metals such as Rb and Cs in the lepidolite ore [18,19]. Therefore, according to the geomorphic characteristics of different regions, the chemical compositions and contents of valuable elements in lepidolite vary to a certain extent.

The color of lepidolite minerals ranges from purple to colorless. They have a pearly luster and stable chemical properties. Lepidolite crystals belong to the monoclinic crystal system and often appear as fine scaly aggregates. The T-O-T-type crystal structure composed of silicon–oxygen tetrahedrons (T) and aluminum–oxygen octahedrons (O) [13] is shown in Figure 5. Al^3+^, Fe^2+^ and Mg^2+^ mainly occupy the octahedral positions in the crystal lattice. Alkali metal cations such as K^+^, Na^+^ and Cs^+^ fill between the unit layers [18,19]. Cations such as Si^4+^ and Al^3+^ mainly occupy the tetrahedral positions in the lattice structure, forming a stable 2:1 type structure [20,21].

#### 2.1.1. Acid Leaching

The acid leaching method involves reacting lepidolite ore with one or more acids in different ratios under certain conditions. The reaction process destroys the inherent structure of lepidolite, converting valuable elements such as rubidium and cesium into soluble salts [18]. These elements enter the liquid phase during the leaching process, and the reaction formula is shown as (1). After acid leaching, through processes such as impurity removal and separation, as well as precipitation separation of the leaching solution, crude products of rubidium, cesium and lithium can be obtained.

(1) $$4{KLi}_{1.5}{Al}_{1.5}{\left[{AlSi}_{3}O_{10}](OH\right)}_{2}+20H_{2}{SO}_{4}\to 2K_{2}{SO}_{4}+3{Li}_{2}{SO}_{4}+5{Al}_{2}{\left({SO}_{4}\right)}_{3}+12H_{4}{SiO}_{4}↓$$

According to the type of acid selected, the acid leaching method is usually divided into the sulfuric acid leaching method and the fluorochemical method. The sulfuric acid leaching method is often combined with roasting and curing techniques, which is called the sulfuric acid roasting/curing leaching method. The sulfuric acid roasting leaching method loosens the structure of lepidolite by roasting it at high temperature. H^+^ in the acid replaces Li^+^, Rb^+^, Cs^+^ and other ions in the mineral phase lattice [17,20]. This transfers Rb and Cs to sulphates, and they are dissolved in the liquid phase during aqueous leaching. In the sulfuric acid curing leaching method, concentrated sulfuric acid undergoes a curing reaction with lepidolite ore at a relatively low temperature, and then Rb and Cs are extracted through water leaching.

**Figure 6:**
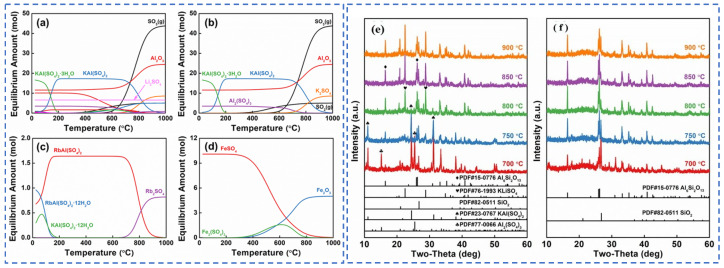
(**a**) Thermodynamic models, (**b**) K, Al, S, (**c**) Rb, and (**d**) Fe and phase changes (**e**,**f**) of each component of lepidolite in different reactions with sulfuric acid [23].

**Figure 7:**
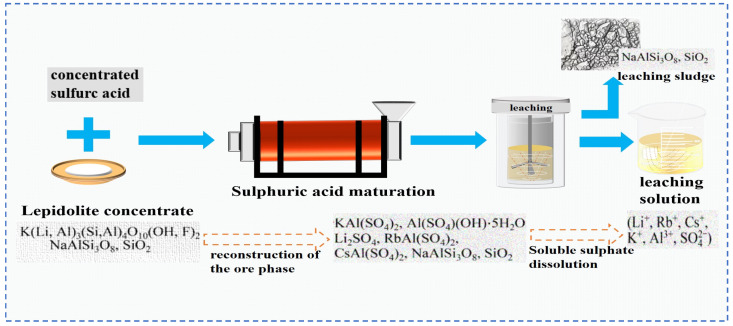
Phase transformation diagram of extraction of rubidium lithium cesium by sulfuric acid curing of lepidolite concentrate [26].

Zhang [22] employed the sulfuric acid roasting-water leaching method to extract valuable elements like Li, Rb and Cs. Their study indicated that during the roasting process, sulfuric acid and lepidolite ore are mixed in a mass ratio of 1.7:1 and roasted at 200 °C for 4 h. Subsequently, the roasted ore is subjected to a water leaching reaction at 85 °C, and the leaching rates of Rb, Cs and Li are found to be greater than 95%. Liu et al. [23] aimed to address the problem in the sulfuric acid roasting-water leaching method, where during the leaching process, lithium, rubidium and cesium are entrained by the iron-aluminum slag in the leaching solution, resulting in losses. They proposed a two-step sulfuric acid roasting method to avoid the generation of iron-aluminum slag during the leaching process and reduce the losses of Li, Rb and Cs from the source. By combining the establishment of a thermodynamic model, performing calculations as shown in Figure 6 and analyzing the changes in components and phases during the reaction between lepidolite and sulfuric acid at different temperatures, the selective extraction of valuable elements can be achieved. An excessively high roasting temperature leads to severe energy consumption. With continuous improvement of the sulfuric acid method, the extraction of rubidium and cesium from lepidolite is gradually carried out under low-temperature and mild reaction conditions. Liu [24] has carried out continuous research on the low-temperature sulfuric acid roasting–leaching process, and systematically investigated the influence between technological factors such as the volume-to-mass ratio of acid to ore and the roasting temperature, as well as the roasting temperature itself. Under the low temperature of 150 °C, lepidolite is mixed with sulfuric acid and roasted for 12 h. Then, it is leached with water at room temperature for 40 min, achieving the synchronous and efficient leaching of lithium, rubidium, and cesium. However, the technological process is time-consuming and causes serious energy consumption losses. In comparison, the sulfuric acid curing-water leaching process promotes a reaction between sulfuric acid and lepidolite at a lower temperature, which greatly reduces energy consumption. Zhang [25] systematically studied the effects of factors such as acid-to-ore ratio, curing temperature, solid-to-liquid ratio and leaching time using the sulfuric acid curing-water leaching process. Results showed that when 70% sulfuric acid with a mass ratio of 1:1 to lepidolite ore was subjected to a curing reaction at 120 °C for 8 h, followed by leaching at 50 °C for 1 h at a solid-to-liquid ratio of 4:1, the leaching rates of Rb, Cs and Li were 88.83%, 90.09% and 91.42%, respectively. Zhang [22,26] studied the sulfuric acid ripening-water leaching process in depth, using kinetic related calculations to investigate the mechanism of substance-phase transformation during the reaction process, and the reaction control factors as shown in Figure 7. After high-temperature curing of lepidolite, mineral phase reconstruction occurs. When the sulfuric acid medium passes through the solid product layer, the process is controlled by internal diffusion. Rubidium, cesium and lithium exist in the leachate in the form of Li_2_SO_4_, RbAl(SO_4_)_2_ and CsAl(SO_4_)_2_, enabling efficient extraction of rubidium and cesium.

Although the sulfuric acid leaching method can effectively extract valuable metals from lepidolite ore under relatively simple and mild conditions, the sulfuric acid consumption is high, and a large amount of alkali is required in the subsequent process to neutralize the residual acid and precipitate the dissolved aluminum. Because lepidolite ore contains approximately 20% Al_2_O_3_, separating it from the leaching mother liquor generates a large amount of Al(OH)_3_ precipitate. During filtration and separation, this precipitate adsorbs and causes loss of rubidium, cesium, etc., resulting in low extraction rates [24].

#### 2.1.2. Fluoride Chemical Method

The Fluoride Chemical Method is an improvement over the sulfuric acid leaching method, using fluoride-containing acid solutions such as HF or HF + H_2_SO_4_ to replace strong acids. HF can effectively break the silicon–oxygen bonds (Si–O) in lepidolite, significantly enhancing the decomposition process of mineral phases. Rubidium (Rb^+^) and cesium (Cs^+^) ions are exchanged into the liquid phase, improving the leaching efficiency of rubidium and cesium elements, with the reaction form as shown in (2) [27,28].

(2) $${KLi}_{1.5}{Al}_{1.5}{AlSi}_{3}O_{10}F_{2}+16HF+5H_{2}{SO}_{4}\to 0.75{Li}_{2}{SO}_{4}+1.25{Al}_{2}{\left({SO}_{4}\right)}_{3}+3H_{2}{SiF}_{6}+0.5K_{2}{SO}_{4}+10H_{2}O$$

**Figure 8:**
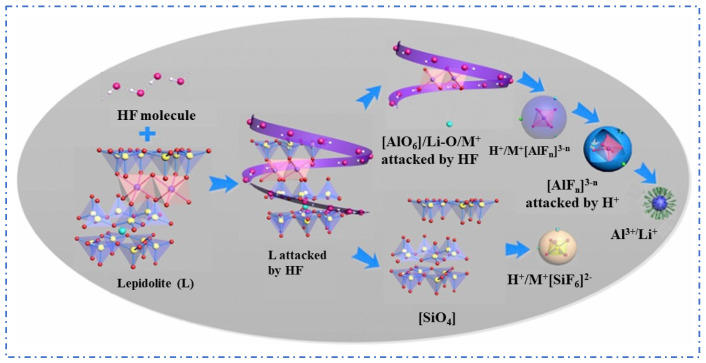
Lepidolite dissolution mechanism [21].

Guo [21] used a mixed solution of HF and H_2_SO_4_ to leach lepidolite and conducted a fundamental theoretical study on the mineral dissolution mechanism during the reaction, as shown in Figure 8. The results show that the mixed acid has an enhanced effect on mineral dissolution, where HF mainly breaks chemical bonds while H_2_SO_4_ accelerates the dissolution reaction and influences the coordination equilibrium between Al^3+^ and F^−^. The optimal process conditions were determined as follows: lepidolite is leached with HF and H_2_SO_4_ at a ratio of 1:2:3.5 at 85 °C for 3 h, which allows for the recovery of over 90% of rubidium and cesium. To reduce economic costs, Guo et al. [29] further improved the process by using a mixture of H_2_SiF_4_ and H_2_SO_4_ and adopted a continuous tubular reactor to enhance the effect of the acid leaching reaction. Leaching lepidolite concentrate with a mixture of H_2_SO_4_ and H_2_SiF_6_ achieves the recovery of 97.6% of Rb and 96.7% of Cs under low-temperature and short-time conditions. Although the Fluoride Chemical Method reduces acid concentration and demonstrates excellent recovery efficiency for rubidium and cesium, the introduction of a large amount of fluoride ions not only increases the difficulty of subsequent processes but also poses a risk of environmental pollution, thus limiting its industrial value.

#### 2.1.3. Alkaline Leaching Method

The alkaline leaching method involves uniformly mixing one or more alkalis such as sodium hydroxide (NaOH) and aluminum hydroxide (Al(OH)_3_) with lepidolite ore, followed by a leaching reaction. The leaching agent reacts with the mineral lattice, promoting the dissociation of alkali metal elements such as rubidium (Rb) and cesium (Cs) from the mineral structure. These elements ultimately enter the leaching solution in the form of rubidium hydroxide (RbOH) and cesium hydroxide (CsOH).

**Figure 9:**
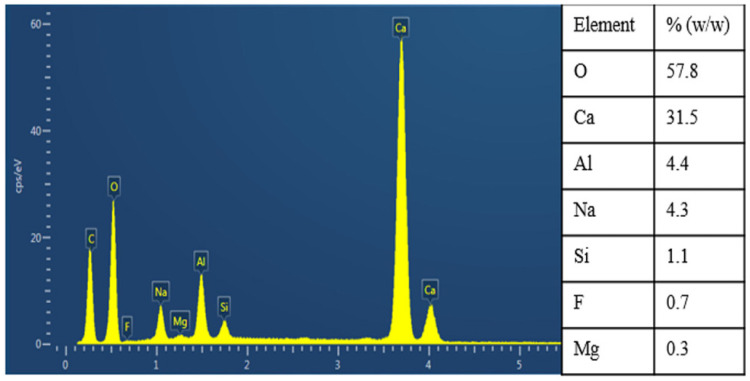
Analysis of sediment during liquid purification [14].

Wang [30] adopted the sodium hydroxide alkaline leaching method to investigate the disruption of lepidolite structure by a single alkali and the dissolution behavior of valuable elements such as Rb^+^, Cs^+^ and Li^+^. Under the optimal process conditions of leaching reaction with a 50% mass fraction NaOH solution at a mass ratio of 3.5:1 to lepidolite ore at 190 °C for 4 h, cation resin was used to achieve one-step recovery of lithium, rubidium and cesium. With further advancement of research, some researchers have found that compared with using a single alkali as the leaching agent to extract rubidium and cesium from lepidolite, mixed alkalis can more completely disrupt the mineral structure of lepidolite, leading to higher dissolution rates of valuable elements such as rubidium, cesium and lithium during the leaching process. James Mulwanda [14] employed a mixed alkali leaching method using (NaOH + Ca(OH)_2_) under high temperature and pressure conditions. Through techniques such as filtration, washing, precipitation, and separation, they achieved the simultaneous recovery of Rb, Cs, Li and K as hydroxides, with leaching rates of 96%, 90%, 94% and 98%, respectively. Purification and analysis of the components in the residual leaching solution are shown in Figure 9. By recovering residual rubidium, cesium, lithium and remaining alkali, resource recycling is achieved, which greatly reduces production costs. Huang [31] introduced calcium oxide on the basis of the alkaline method, adopted a mixed alkali leaching method using (NaOH + CaO), carried out alkali leaching in a high-pressure reactor and performed solid–liquid separation and slurry washing separation on the reactants so as to achieve the efficient extraction of valuable elements from lepidolite. Under optimal reaction conditions, the dissolution of impurities was reduced, enabling the one-step preparation of products such as lithium carbonate.

**Figure 10:**
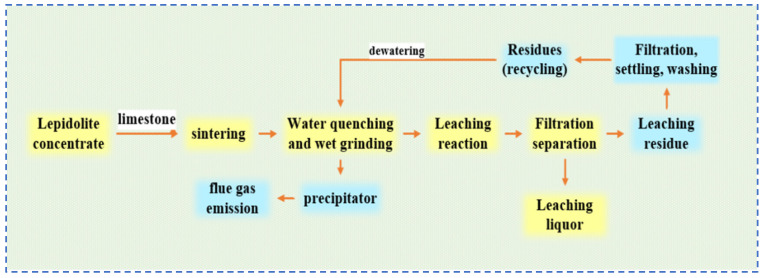
Flow chart of extraction process of rubidium, lithium and cesium resources from lepidolite [32].

Sun [32] conducted an in-depth study on the common process flow for extracting rubidium and cesium from lepidolite, as shown in Figure 10. They analyzed the distribution of rubidium and cesium resource losses during the process and carried out a detailed theoretical analysis of the causes of these losses. Rubidium and cesium losses are mainly concentrated in sintering losses, flue gas entrainment, water leaching residue, and residual liquid attached to the residue during the roasting process. Optimizing key processes such as roasting can reduce resource loss by 20–35%, thereby increasing the comprehensive recovery rate of rubidium, cesium and lithium by approximately 80%. This effectively reduces production costs and significantly enhances output value and benefits. Sun [32] provided theoretical guidance for improving the recovery rate of rubidium and cesium resources in lepidolite, laying a foundation for the development of new processes. Wang [33] treated lepidolite using an alkaline leaching method with Ca(OH)_2_ and Na_2_CO_3_. To avoid the formation of fluorides, steam roasting is used to alter the mineral structure. Through this process, lepidolite is transformed into aluminosilicate and leucite, resulting in a loose, porous and highly reactive mineral material with an optimized structure. During the leaching process, Ca(OH)_2_ first reacts with Na_2_CO_3_ to generate a strong base (NaOH). The Na^+^ then undergoes a displacement reaction with Rb^+^, Cs^+^ and Li^+^ in the mineral lattice, converting them into water-soluble hydroxides (RbOH, CsOH and LiOH) that enter the liquid phase. Simultaneously, the dissolution of impurity elements such as Al_2_O_3_, SiO_2_ and F^−^ is significantly inhibited. The reaction principle equations are shown as Equations (3)–(6). Through multi-faceted research, it was determined that under the optimal conditions of roasted material—Ca(OH)_2_:Na_2_CO_3_ = 10:9:2, leaching temperature at 140 °C, and reaction for 3 h,—efficient recovery of rubidium, cesium and lithium can be achieved.

Na_2_CO_3_ + Ca(OH)_2_ → 2NaOH + CaCO_3_ (3)

Me_2_O + Al_2_O_3_ + 2SiO_2_ + 2NaOH + 2H_2_O → Na_2_O·Al_2_O_3_·2SiO_2_·2H_2_O + 2MeOH (4)

Na_2_O·Al_2_O_3_·2SiO_2_·2H_2_O + Ca(OH)_2_ → CaO·Al_2_O_3_·2SiO_2_·2H_2_O + 2NaOH (5)

K_2_O·Al_2_O_3_·4SiO_2_ + Ca(OH)_2_ + 2H_2_O → CaO·Al_2_O_3_·4SiO_2_·2H_2_O + 2KOH (6)

In the equation, Me represents Rb, Cs, Li, Na and K.

The alkaline method offers high simultaneous recovery rates for valuable elements such as rubidium, cesium and lithium, featuring a simple process, low impurity ion dissolution rate, and low corrosion to equipment. Additionally, the intermediate product in the experiment—aluminosilicate sol—can be directly sold, enhancing the added value of the products. However, the high concentration of the alkaline solution requires attention on subsequent treatment and recovery issues. Additionally, impurity ions from the mineral remain in the solution, which may cause coprecipitation during evaporation and precipitation, thereby reducing the purity of rubidium and cesium salts.

#### 2.1.4. Salt Roasting-Leaching Method

The essence of the salt roasting process is that one or several mixed salts from sulfates, chlorides, and carbonates are mixed with lepidolite minerals in proportion for roasting. This process transforms or disrupts the mineral structure, enabling ion exchange reactions between Rb^+^, Cs^+^ and Li^+^ in lepidolite and alkali metal ions in the salts. After the leaching reaction process, these ions exist in the solution in the form of soluble salts.

Zhang [34] adopted the sulfate roasting-water leaching method, mixing ferric sulfate with lepidolite, in which both gas–solid and liquid–solid reactions simultaneously occur in the reaction system. The cyclic phase transition process of pyrosulfate and sulfate achieves the alternating activation effect of the two salts on lepidolite, where the low-melting-point pyrosulfate significantly promotes the decomposition reaction of lepidolite. Through exploration of the process conditions, the optimal process conditions were finally determined as follows: the mass ratio of ferric sulfate to lepidolite is 2:1, roasting at 675 °C for 90 min, ultimately achieving simultaneous extraction of Rb, Cs and Li. During high-temperature roasting, lepidolite is prone to sintering into blocks. To address this phenomenon, Guo et al. [35] adopted a mixed sulfates roasting-leaching method. Potassium sulfate, calcium sulfate and barium sulfate mixed salts are used to roast with lepidolite at 900 °C for 1 h. After roasting, the minerals are leached with dilute hydrochloric acid for 1 h. The leaching rate of lithium resources exceeds 90%, but the leaching rates of rubidium and cesium are low. Therefore, Guo [36] made improvements under these experimental conditions by replacing part of the calcium sulfate with barium sulfate and optimizing the ratio between the ore and the sulfuric acid mixed salt. Under the same experimental conditions, the improvement of the reagent has significantly increased the leaching rate of rubidium and cesium. This process achieves leaching under mild experimental conditions and reduces reagent consumption compared to traditional sulphate methods, thereby more effectively reducing environmental pollution hazards. This represents a certain progressive significance compared to the traditional sulfuric acid method.

**Figure 11:**
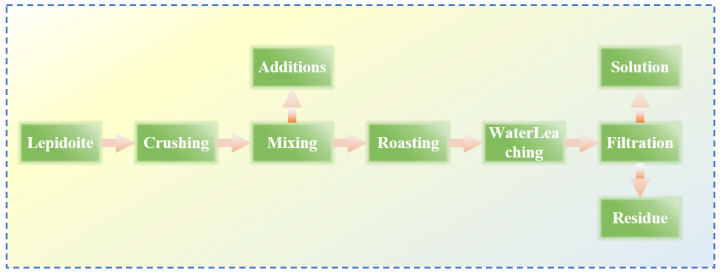
Flow chart of sulfation–roasting–leaching process of lepidolite ore [37].

**Figure 12:**
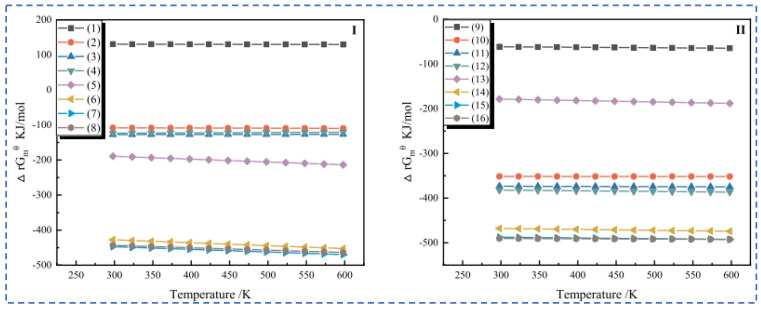
(**I**,**II**) Curve analysis diagram of HSC model in roasting process [39].

Yan [37] made improvements on the basis of the original sulfate roasting-water leaching method by reacting a mixture of sulfates and oxides with lepidolite, thereby reducing the consumption of sulfates. Lepidolite:Na_2_SO_4_:K_2_SO_4_:CaO are mixed uniformly at a mass ratio of 1:0.5:0.1:0.1, roasted at 850 °C for 0.5 h, and then subjected to a water leaching reaction at room temperature with a liquid–solid ratio of 2.5:1 for 0.5 h. Through processes such as filtration, separation, washing, and precipitation, valuable metals are recovered from the residue to the greatest extent. The reaction flow is shown in Figure 11, achieving simultaneous recovery of Rb, Cs, Li and K. Yan [38] adopted the chloride roasting-water leaching method and conducted an in-depth study on the effects of various process parameters such as roasting time and temperature of the chlorinating agent. Lepidolite was roasted with NaCl and CaCl_2_ at a mass ratio of 1:0.6:0.4 at 880 °C for 30 min, followed by a water leaching reaction at 60 °C for 30 min. The leaching rates of Rb and Cs reached 93.6% and 93.01%, respectively. This process is simple and achieves the advantages of simultaneous leaching of Li, Rb and Cs with high leaching efficiency and less waste residue. Wang [39] conducted an in-depth study on the mixed salt roasting-water leaching method and used a mixture of sulfates and chlorides. The mechanism of the roasting process was calculated and analyzed using the HSC thermodynamic model as shown in Figure 12, to explore the influence of the roasting process on the water leaching process. It was determined that a mixed salt of Na_2_SO_4_ and CaCl_2_ was used as the reaction agent for roasting with the mineral. Thermodynamic analysis shows that Na_2_SO_4_ cannot directly interact with (Li, Rb and Cs) oxides. Instead, SiO_2_ and Al_2_O_3_ initiate highly chemically selective ion exchange between Li^+^, Rb^+^ and Cs^+^ in the ore and Na^+^ in Na_2_SO_4_ and Ca^2+^ in CaCl_2_, achieving simultaneous extraction of Li, Rb, Cs, Na and K from lepidolite.

The salt roasting-water leaching method exhibits high ore adaptability depending on the type of salt used, enabling the processing of valuable metal resources such as rubidium and cesium in ores with different grades. During the roasting process, it reduces the formation of soluble salts of aluminum, minimizing the loss of rubidium and cesium caused by impurity ions in subsequent impurity removal processes via precipitation. In addition, this method features short process time and high recovery rates of rubidium and cesium. However, the high roasting temperature leads to severe energy consumption in the process flow, the process conditions are relatively harsh, and the exhaust gas generated during the experiment is difficult to treat, which is not conducive to environmental safety.

### 2.2. Zinnwaldite

The chemical formula of zinnwaldite is (KLiFeAl[AlSi_3_O_10_](F, OH)_2_), which contains 11.5% Fe_2_O_3_ and 1.1% MnO. As a transitional mica group mineral, it is currently mainly considered for lithium resource extraction and recovery from solid concentrates, with fewer processes considering the recycling and utilization of rubidium and cesium resources therein.

In existing processes, inorganic reagents containing Ca^+^ are often added to zinnwaldite as combustion aids to better dissolve the mineral during the mineral roasting process. Combined with the water leaching process, metal resources are recovered through processes such as filtration separation, purification and impurity removal, and solid–liquid separation. As a transitional product of lepidolite, zinnwaldite has a similar structure to lepidolite, so the extraction processes are alike. Zeng [40] conducted a comparative study on the chloridizing roasting-water leaching process and direct acid leaching method for zinnwaldite. There are significant differences in the action mechanisms of different inorganic roasting agents containing Ca^2+^. The carbon dioxide produced by the decomposition of CaCO_3_ cannot effectively break the mineral structure. The practical application of CaSO_4_ is limited due to its high decomposition temperature. In contrast, CaCl_2_ shows excellent decomposition performance because of its high chlorine content. At 900 °C, CaCl_2_ reacts with biotite at the liquid–solid interface, exhibiting extremely fast reaction kinetics, and achieves efficient recovery of rubidium and cesium with extraction rates exceeding 90%. However, RbCl formed by the chlorination reaction is prone to volatilization at high temperatures, reducing the recovery rate of rubidium. Therefore, Zeng [40] conducted in-depth research on the reaction mechanism of acid leaching. The iron elements in zinnwaldite promote the formation of pyrite, which significantly increases the difficulty of dissolving alkali metal elements. However, in the acid leaching process at high temperatures, rubidium, cesium and potassium in the acid leaching solution exhibit high purification degrees. Guo [41] adopted the chloridizing roasting-ultrasound-assisted leaching process, which significantly shortened the leaching time. Under ultrasonic action, the negatively charged parts of polar water molecules surround metal ions, while the positively charged parts surround chloride ions. Rubidium and cesium leave the crystal lattice in the form of chlorides and enter the solution. Under the optimal conditions of a liquid–solid ratio of 4:1, an ultrasonic power of 100 W, a leaching temperature of 60 °C, and a leaching time of 20 min, efficient recovery of rubidium and cesium can be achieved. Currently, there are relatively few studies on the recovery of metal resources from zinnwaldite, but it has great potential for marketization.

## 3. Salt Lake Brine and Lithium Ore Leachate

Salt lake brines are rich in rubidium and cesium reserves, but approximately 80% of these rubidium and cesium resources have low mining value due to limitations imposed by their occurrence characteristics. Due to the fact that rubidium and cesium mainly occur in combined states and their concentrations are low (with an average rubidium content of 10.8 mg/L and cesium content of 0.034 mg/L) congeneric elements with similar chemical properties (Li, Na and K) [42] coexist with rubidium and cesium in salt lake brines and geothermal water to form multicomponent coexisting systems, posing certain technical barriers to the highly selective separation of rubidium and cesium metals. Efficient green extraction technology serves as the key research direction for the comprehensive extraction of rubidium and cesium resources from salt lake brines.

In hard-rock minerals, attention is often only paid to the extraction of lithium resources, but the rubidium and cesium resources contained in the leachate remaining after lithium extraction (lithium extraction mother liquor) are frequently overlooked [43], leading to resource waste. Therefore, the efficient recovery of rubidium and cesium resources from lithium extraction mother liquor, reduction of resource loss and enhancement of production capacity have become core issues in resource recycling.

Whether for salt lake brine or lithium extraction mother liquor, methods such as precipitation, solvent extraction, and ion exchange are used to extract and recover rubidium and cesium resources from the solution.

### 3.1. Precipitation Method

The precipitation method involves reacting certain precipitants with metal ions in a solution. By adjusting process conditions such as precipitant concentration and acidity-alkalinity, the reaction is induced to form precipitates, thereby achieving the separation of rubidium and cesium ions or selective extraction and recovery of rubidium and cesium ions [44]. Precipitants are rich and diverse in types, and are mainly classified into compounds such as heteropoly acids, complex salts, halides and sulfate salts according to reagent categories. At present, the main research on precipitants for recovering rubidium and cesium ions focuses on silicomolybdic (tungstic) acid, potassium iodobismuthate, chloroplatinic acid, stannic chloride, antimony trichloride, iodine monochloride, aluminum sulfate, etc., as specifically shown in Table 4. This technology is worth adopting due to its simplicity, ease of use, and suitability for large-scale applications. To further enhance the recovery rate and purity of rubidium and cesium ions, the precipitation method is often combined with other technologies for joint application.

Although the precipitation method exhibits extremely high recovery efficiency for rubidium and cesium in salt lake brine, the low content of rubidium and cesium in salt lakes, coupled with the high cost, complex reactions, and poor stability of the precipitated compounds in the precipitation method, make it unsuitable for large-scale industrial production. Therefore, the precipitation method was more common in the early industrial production of rubidium and cesium but has seen less market application in recent years.

### 3.2. Solvent Extraction Method

The solvent extraction method is a separation technique based on the liquid–liquid interface. It mainly utilizes the difference in solubility of Rb^+^ and Cs^+^ ions in two immiscible (or slightly miscible) solvents to achieve selective extraction. The essence of extracting rubidium and cesium from salt lake brine by the solvent extraction method is to add an organic extractant to the aqueous phase containing rubidium and cesium, so that the target ions form a hydrophobic complex with the extractant. Since the organic phase and the aqueous phase are immiscible, the complex will transfer to the organic phase, thereby achieving separation from other ions.

Extractants include phenolic alcohol reagents, crown ethers and dipicrylamine and its derivatives. Among them, crown ethers exhibit broad applicability in the extraction and separation of alkali metal ions due to their unique cyclic cavity structures. The extraction capability is related to its structure. Through the differences in the types of substituents and cavity sizes of crown ethers, the selective separation of cations with different volumes can be achieved. For Rb^+^ and Cs^+^, the commonly used crown ethers are 18-crown-6 (cavity size 1.4–1.7 Å) and 21-crown-7 (cavity size 1.7–2.2 Å), respectively. Traditional crown ether systems are mostly used in strongly acidic environments, and their high economic cost has greatly restricted their industrialization process. However, in recent years, through the composite modification of crown ethers with polymer carriers and functional nanomaterials, traditional organic solvents and other technologies have been gradually replaced in fields such as new energy and strategic metal resource recovery. The extraction of Cs^+^ and Rb^+^ by dipicrylamine (hexanitrodiphenylamine) requires operation under alkaline conditions, and the remaining solution will cause environmental pollution. Therefore, it is rarely used at present.

The current commonly used extractants are phenolic reagents (BAMBP/t-BAMBP) [45]. Through the back-extraction method, Cs^+^ and Rb^+^ undergo displacement with the H^+^ of phenol, and the Cs/Rb complexes enter the organic phase, as shown in equation (7). Phenolic reagents can also perform stepwise for continuous extraction reactions of alkali metal elements by regulating process conditions, based on their extraction capability for alkali metals (Cs^+^ > Rb^+^ > Ca^2+^ > K^+^ > Li^+^ > Mg^2+^ > Na^+^). With its high selectivity and high salt tolerance, this technology demonstrates industrial potential in the field of rubidium and cesium extraction from salt lake brine. Its key lies in balancing the contradiction between reagent stability and separation efficiency.

(7) $${Cs}^{+}/{Rb}^{+}+4ROH-Cs/RbOR\cdot {\left(ROH\right)}_{3}+H^{+}$$

LIU [46] used a sulfonated kerosene solution of the extractant t-BAMBP as the organic phase to carry out a contact reaction with salt lake brine, and extracted Rb^+^ and Cs^+^ by the back-extraction method. The optimal process conditions are as follows: contacting a 1.0 mol/L sulfonated kerosene solution of t-BAMBP with an aqueous phase of 1 mol/L alkalinity, adjusting the oil/water ratio to 1:1, washing three times with NaOH lye and performing five-stage countercurrent extraction. This achieves extraction rates of rubidium and cesium as high as 95.04% and 99.80%, respectively. Chen [47] used t-BAMBP and ammonia as the extractant and stripping agent, respectively, to react with high-concentration salt lake brine for the sequential continuous recovery of Cs and Rb. Through exploration and improvement of experimental conditions, it was determined that when the solution pH was adjusted to 8 at 35 °C, 0.1 M t-BAMBP was used as the extractant to react with brine. The organic phase-to-aqueous phase ratio was first adjusted to 0.3, and after a 3 min contact reaction, the ratio of organic phase to aqueous phase was adjusted to 2. Then, upon cooling to 25 °C, 1M ammonia was added for the separation of Cs. The separation conditions for rubidium (Rb) are as follows: adjusting the pH to 12 at 5 °C, reacting 0.5 M t-BAMBP extractant with brine for 15 min to achieve separation of Rb and K, then adding ammonium carbonate to recover Cs and Rb. Finally, rubidium with a purity of 98.0% and cesium with 98.9% purity are obtained. PANG Deng-ke [48] selected t-BAMBP as the extractant by comprehensively considering environmental and economic impacts. The presence of K^+^ and Mg^2+^ increases the difficulty of rubidium and cesium extraction. A double precipitation system is utilized to separately precipitate K_2_SO_4_ crystals and Mg(OH)_2_ precipitates, which can be directly sold as products to reduce economic costs. By comprehensively optimizing process conditions such as pH, contact reaction time and gas phase ratio, 100% extraction of Cs^+^ is achieved, the extraction efficiency of Rb^+^ reaches as high as 85.8%, and 8.76% of K^+^ is simultaneously extracted and recovered. This process can achieve the extraordinary enrichment of rubidium and cesium with only 2 min of contact reaction. However, it requires strong alkaline conditions, and there is still room for improvement in reducing acid base consumption during the extraction and separation procedures.

**Figure 13:**
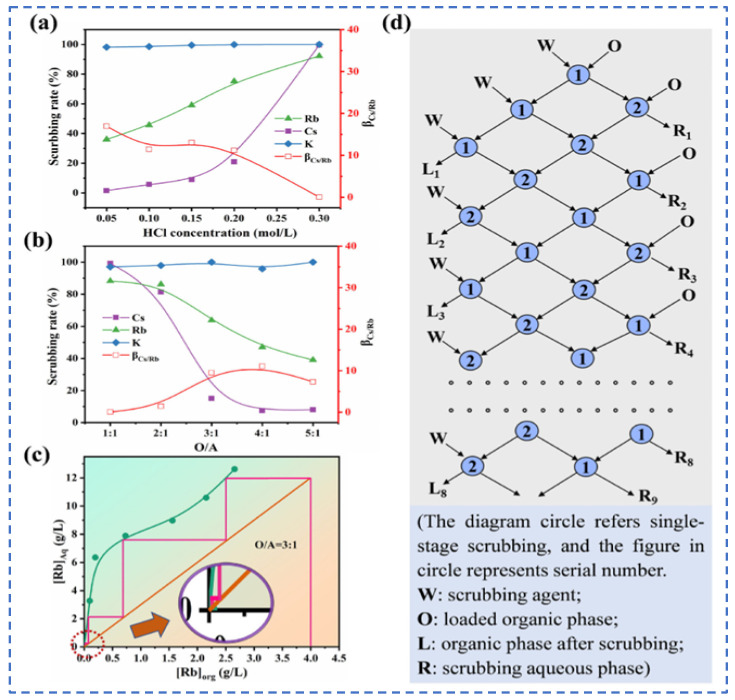
Influence of process conditions on extraction rate of Rb+ and Cs+ (**a**,**b**), (**c**) Scrubbing isotherm and multi-stage washing scheme (**d**) [49].

**Figure 14:**
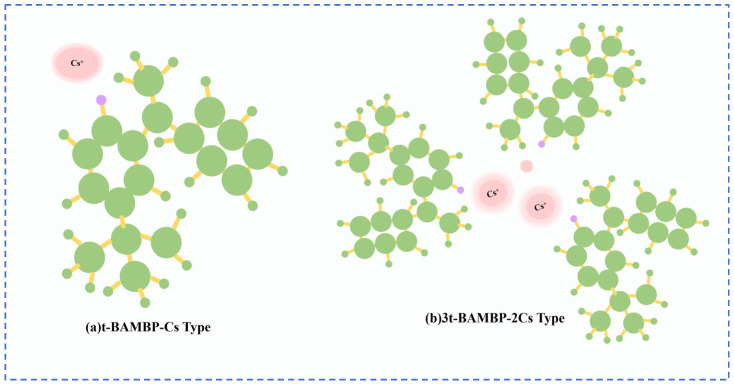
Molecular cluster model [50].

Lv et al. [49] used t-BAMBP as an extraction agent to extract rubidium and cesium resources from lithium extraction mother liquor, and conducted a systematic study on the process conditions. Finally, the optimal process conditions were determined as follows: contacting t-BAMBP with a concentration of 1 mol/L with the feed solution having an alkalinity of 10 g/L at a phase ratio of 1:4 for a contact reaction time of 1 min. Multistage countercurrent washing of the organic phase was carried out as shown in Figure 13. Under this condition, 99% of Cs in the organic phase was stripped by acid, while the washing efficiency of Rb reached 99.83%. However, due to the high consumption of reagents in this process leading to excessively high costs, Zhu [50] adopted an extraction system of t-BAMBP + sulfonated kerosene + cyclohexane, and analyze the stability of rubidium and cesium extraction processes in low-concentration lithium solutions by incorporating thermodynamic principles. A molecular cluster model was established to analyze the molecular morphology during the reaction process. Rubidium and cesium form stable 3t-BAMBP-2Cs-type molecular clusters with t-BAMBP, ensuring the stable presence of rubidium and cesium in the organic phase, as shown in Figure 14. Under optimal reaction conditions of a temperature of 25 °C, a pH of 12.5, an extraction phase ratio of 1:1 and a contact reaction time of 5 min, the extraction rate of Cs reached 99.52%, and the stripping rate of Rb reached 96.69% when the lithium extraction mother liquor was reacted with 0.5 mol/L extractant. Zhang [51] studied the recovery of cesium resources from the leachate of lepidolite after the sulfuric acid roasting process, and adopted a new process of crystallization–roasting–dissolution–solvent extraction to extract cesium from the leachate. When the temperature of the leachate is lowered, rubidium and cesium precipitate in the form of mixed alum. The mixed alum is decomposed into Cs_2_SO_4_ and Rb_2_SO_4_ by calcination at 900 °C. During the water dissolution reaction, rubidium and cesium are transferred into a mixed sulfate solution, which is then subjected to contact extraction with t-BAMBP. After washing, stripping, evaporation and crystallization, cesium sulfate is recovered with a purity as high as 98.47 wt%.

**Figure 15:**
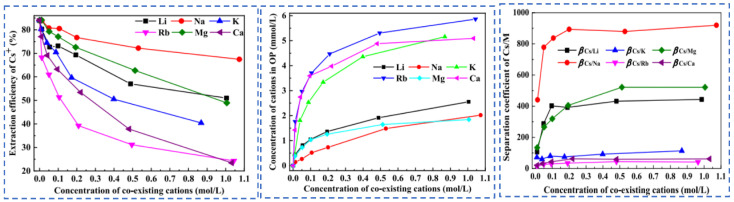
Effects of coexisting cations on Cs^+^ extraction [52].

**Figure 16:**
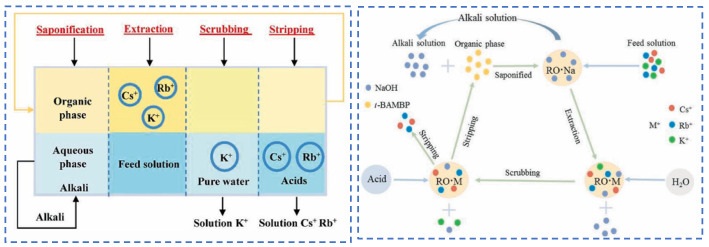
The extraction process of Cs^+^ and Rb^+^ separation and the transfer of ions in different stages [53].

Due to the extremely high consumption of traditional solvent extraction methods leading to excessive costs, Cheng [52] considered environmental and economic impacts and proposed a synergistic system of two extractants (t-BAMBP-D2EHPA) for the extraction and recovery of Cs resources from salt lake brine. The coexisting ions (K^+^, Li^+^, Mg^2+^ and Na^+^) in salt lake brine significantly inhibit the extraction efficiency of Cs^+^, as shown in Figure 15. In the synergistic system, the ion extraction sequence is dominated by t-BAMBP, which preferentially extracts Rb^+^ and Cs^+^. Using a three-stage countercurrent extraction process, the extraction efficiency of Cs^+^ reaches 92.6%. During the extraction and stripping processes with t-BAMBP, inevitable large consumption of acids and bases occurs. In response, Dengke Pang [53] conducted in-depth research on the extraction mechanism of t-BAMBP and proposed a process using saponification, extraction, scrubbing, and stripping to recover rubidium and cesium resources from brine. Saponification reaction is carried out on the organic phase, where the phenolic hydroxyl groups in the extractant serve as binding sites for alkali metal ions. The hydrogen in the phenolic hydroxyl groups dissociates from t-BAMBP, promoting the preliminary extraction of Cs^+^ and Rb^+^. During the further extraction process, Cs^+^ and Rb^+^ form a complex MOR·3ROH (M = Cs^+^, Rb^+^, K^+^) in the organic phase. Under the washing and stripping processes, the MOR·3ROH complex dissociates in an acidic environment, enabling the separation of Cs^+^ and Rb^+^ from the mixed phase. After the reaction, protons (H^+^) in the solution bond with the phenolic hydroxyl groups, as shown in Figure 16. This regenerates the extraction capability of the t-BAMBP extractant, enabling its cyclic reuse and significantly reducing the environmental pollution caused by the extractant.

Although the solvent extraction method is widely used for the separation of rubidium and cesium due to its high selectivity and ease of operation, it generates highly toxic secondary organic pollutants. Regarding the environmental degradation and toxicity caused by volatile organic solvents, researchers have used room-temperature ionic liquids (RTILs) [54], which are considered more environmentally friendly and have significantly reduced environmental hazards.

### 3.3. Adsorption

Adsorption realizes the selective recovery of rubidium and cesium ions by utilizing the characteristics of rubidium and cesium ions as well as the solution. Its essence lies in the use of ion exchangers to come into contact with the liquid, triggering adsorption and ion exchange reactions, so that rubidium and cesium ions can be directly separated from the solution. Currently, ion exchange adsorbents are mainly classified into organic ionic types and inorganic ionic types. Organic ion adsorbents are mainly composed of ion exchange resins and chelating resins, which are susceptible to interference from high-valent metal ions and have low industrial value. However, it is worth noting that ion-imprinted polymers using crown ethers as ligands have significantly enhanced the adsorption effect on Rb. Through the pre-assembly of “ligand-metal ion-functional monomer”, cross-linking polymerization and template removal, adsorption sites with “memory function” are finally formed, which essentially improves the selectivity and adsorption performance for Rb^+^. Liu [55] et al. used benzo-12-crown-4-ether (B12C4) as a ligand to prepare a novel rubidium (I) ion-imprinted material. It realizes the rapid and highly selective adsorption of rubidium ions. In addition, it still has good stability and reusability after multiple uses. Hashemi [56] et al. used dibenzo-21-crown-7 as the selective crown ether to prepare a nanoscale rubidium ion-imprinted polymer with nanometer-scale particle size. Due to factors such as the relative size of the cation and the ligand cavity, the number of ligand binding sites, and their specific arrangement, the material exhibits high selectivity for rubidium ions, but further improvements are needed to address competing ions. Xu [57] et al. prepared a novel dual-functional magnetic ion-imprinted polymer (Fe_3_O_4_@SiO_2_@IIPs) using 12-crown-4 (12C4) and 18-crown-6 (18C6) as functional monomers. The polymer showed excellent adsorption performance and selectivity for Rb and Li, while exhibiting low competitiveness for Na, K, and Mg. It still maintains a high adsorption capacity after multiple cycles of use. Compared with traditional adsorbents, it enables specific recognition of rubidium ions, exhibits excellent stability and adsorption performance across a wide pH range, and shows no significant attenuation in adsorption capacity after multiple reuse cycles. It shows certain research potential in the application of rare metal recycling, heavy metal wastewater treatment and other fields.

Inorganic ion exchange adsorbents are based on cations. In ion exchange adsorbents, positively charged ions exchange with specific ions in the liquid, and the target ions are recovered in the ion exchange agent. Inorganic ion exchangers mainly include zeolites, heteropolyacid salts, polyvalent metal phosphates, ferrocyanides and ferricyanides, etc.

**Figure 17:**
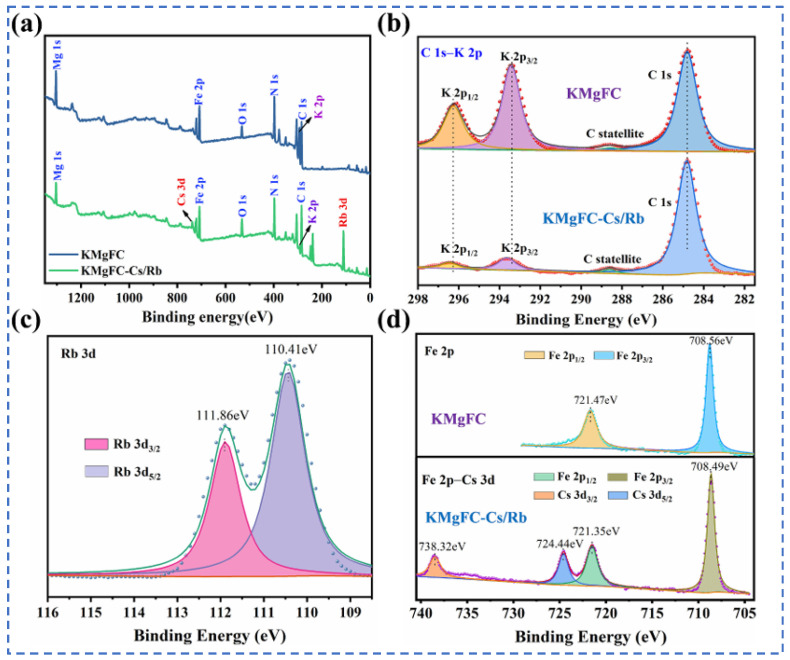
xps diagram of major ions such as rubidium, cesium and potassium before and after adsorption (**a**) adsorption XPS wide-scan spectra; XPS fine spectra of (**b**) C 1s-K 2p, (**c**) Rb 3d, and (**d**) Fe 2p-Cs 3d [60].

**Figure 18:**
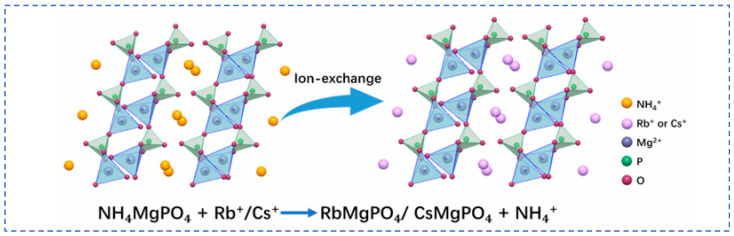
Mechanism of Rb+ and Cs+ ion exchange [61].

**Figure 19:**
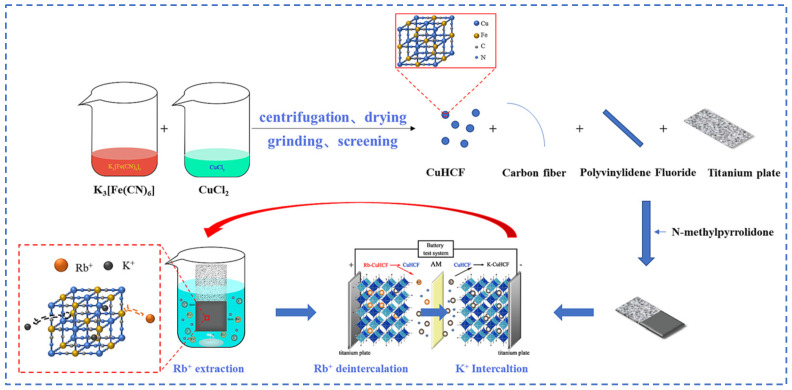
Flow chart of rubidium extraction process [63].

Lian [58] studied polyvalent metal phosphate ion exchange adsorbents, prepared a novel molybdophosphate composite system, and evaluated its capability to recover rubidium and cesium from aqueous solutions. After chemical adsorption of rubidium and cesium ions in water, the adsorbent can be directly regenerated and reused after washing with ammonium nitrate solution, while the extraction efficiency of Rb^+^ and Cs^+^ remains above 90%, showing promising industrial prospects. Qian [59] studied titanosilicate-based ion-exchange adsorbents. Two types of modified potassium titanosilicate were synthesized as adsorbents to extract rubidium and cesium from the residual solution after lithium extraction from lepidolite. The synthesized biomass-derived carbon adsorbent has a mesoporous structure and exhibits good direct adsorption capacity for Rb^+^ and Cs^+^. Ion exchange occurs between K^+^ in the interlayer of the adsorbent and Rb^+^/Cs^+^ in the solution. Through the simultaneous action of adsorption and ion exchange reactions, nearly 100% extraction and recovery of Rb^+^ and Cs^+^ ions are achieved. Lv [60] studied ferrocyanide (KMgFC). Utilizing the high selectivity of ferrocyanide for Rb^+^ and Cs^+^, under the optimal reaction conditions of pH 8.1, temperature 80 °C, and reaction time 40 min, the extraction rates of Rb^+^ and Cs^+^ reached 90.7% and 97.9%, respectively, as shown in Figure 17. Liu [61] and others conducted in-depth research on phosphates. They made a self-prepared ion-exchange adsorbent (ammonium magnesium phosphate) come into contact and react with brine and carried out an in-depth exploration of the reaction mechanism as shown in Figure 18. The adsorption capacities of Rb^+^ and Cs^+^ decrease with increasing temperature. By utilizing reaction enthalpy and Langmuir isotherms, the microcosmic changes of the reaction were explored. In the adsorbent, NH_4_^+^ undergoes monolayer adsorption with Rb^+^ and Cs^+^ in a 1:1 ratio, demonstrating extremely excellent adsorption performance. Wang et al. [62] combined Fe_3_O_4_ with phosphate to fabricate a novel ion-exchange adsorbent (Fe_3_O_4_@ZIF-8@AMP), which exhibits enhanced magnetic properties compared to traditional molybdate-based ion exchange adsorbents. At 25 °C, when adjusting the pH to 6.7, the ion exchanger reacts with brine containing coexisting ions such as K^+^, and only takes 17 s to undergo ion exchange reactions with Rb^+^ and Cs^+^, achieving selective adsorption for the efficient recovery of Rb^+^ and Cs^+^. The novel ion exchanger reacts rapidly and exhibits a high recovery rate, but its adsorption capacity is limited. Therefore, Zhang [63] considered introducing basic electrochemistry on the basis of traditional adsorption methods and progressively proposed combining ion-selective exchange reactions with electrochemical deintercalation to extract rubidium from salt lakes using copper hexacyanoferrate (CuHCF). CuCl_2_ and K_3_Fe(CN)_6_ are precipitated in one step to form CuHCF, which is attached to the electrode. Electrochemically catalyzed ion exchange reactions occur for K^+^ and Na^+^, whereby K^+^ is intercalated into the mineral while Rb^+^ is stripped from the anode into the solution. The process is shown in Figure 19. This method superimposes the electrochemical method onto the traditional adsorption method to directly obtain rubidium resources from salt lakes, realizing selective extraction of resources without generating other wastes.

The adsorption method is widely used in the extraction of rubidium and cesium resources from solutions such as salt lake brines and waste liquids, especially demonstrating significant advantages in the enrichment and separation of low-concentration rubidium and cesium. Compared with other methods, the adsorption method demonstrates stronger application potential by virtue of its remarkable advantages of simple operation, streamlined process flow, and high recovery rate of rubidium and cesium ions. Although issues such as adsorption capacity and stability still need to be gradually addressed through material innovation and process optimization, it is showing enormous application prospects in the field of rare metal recycling along with technological development.

## 4. Silicate Minerals

### 4.1. Rubidium Extraction from Pollucite

Pollucite is the main cesium ore. Its chemical composition is Cs[AlSi_2_O_6_]·nH_2_O, and its crystal structure belongs to the isometric system of silicate minerals [64]. It mainly occurs as associated minerals in lithium-rich granitic pegmatites containing lithium-bearing mica or petalite crystals [65]. Most Pollucite contains 5 to 32 per cent cesium oxide (Cs_2_O), but only 1.5 per cent rubidium oxide (Rb_2_O). It is the most cesium-containing mineral known and is an important mineral ore for the extraction of cesium and rubidium and the production of cesium and rubidium salts.

**Figure 20:**
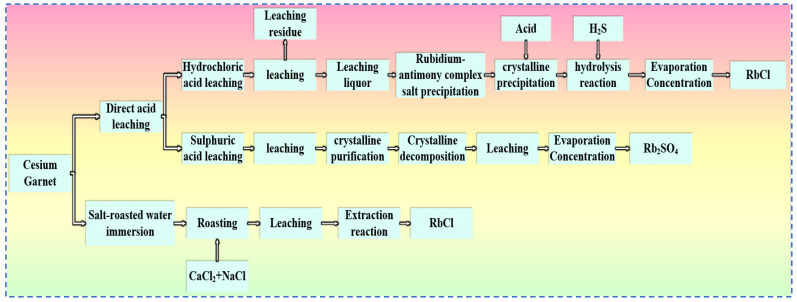
Rubidium–cesium extraction process from cesium garnet.

Currently, acid leaching and salt roasting methods are commonly used to extract rubidium resources from pollucite, as shown in Figure 20. In the acid leaching method, hydrochloric acid or sulfuric acid is typically used to carry out leaching reactions with pollucite, converting rubidium in the ore into a salt form. After crystallization, purification, evaporation, and concentration, the corresponding rubidium salts are obtained. The salt roasting method involves mixing pollucite with salts and roasting the mixture, followed by a water leaching process and then dissolving out rubidium in the form of rubidium salts through a solvent extraction process. The process of extracting rubidium from pollucite by the salt roasting method is relatively mature and shows certain technological prospects. Compared with the acid leaching method used for extracting rubidium and cesium from lepidolite, the process for extracting rubidium from pollucite is more complex, and the tri-wastes (wastewater, waste gas and solid waste) generated after the reaction cause severe environmental impacts.

#### 4.1.1. Hydrochloric Acid Leaching Method

The hydrochloric acid method for pollucite involves leaching pollucite with hydrochloric acid at a certain temperature. In this process, rubidium in the leaching solution is separated by precipitation. In the leaching solution, the precipitant antimony trichloride is added, causing rubidium in the solution to convert into a rubidium antimonate double salt precipitate. The double salt undergoes hydrolysis after crystallization and purification under acidic conditions. Hydrogen sulfide is then introduced to remove interference from coexisting ions in the solution. Finally, the solution is subjected to evaporation and concentration processes to obtain rubidium chloride precipitate. The hydrochloric acid process has a mature technological flow, excellent decomposition efficiency for pollucite, and is widely used in industrial applications. However, the “three wastes” generated during the process—acid leaching residue, double salt precipitation mother liquor, and hydrogen chloride gas—pose significant treatment challenges and high environmental hazards.

#### 4.1.2. Sulfuric Acid Leaching Method

The sulfuric acid leaching method has a mature and complete technological process, and its process flow is simpler than that of the hydrochloric acid method. The main steps are as follows: First, sulfuric acid is uniformly mixed with pollucite and heated for leaching. In this process, rubidium and cesium ions in the solution are converted into alkali metal sulfates, which continuously transform into alum-like phases until rubidium is completely converted into rubidium alum precipitate. The precipitated crystals are purified, and the purified material undergoes further decomposition leaching to selectively recover Rb. Finally, rubidium sulfate product is obtained after evaporation and concentration. In the sulfuric acid method [66], rubidium is converted into rubidium alum, which has a higher solubility in acid solutions at the same temperature than the rubidium antimonate double salt in the hydrochloric acid method. Thus, the metal recovery rate of the sulfuric acid method is higher than that of the hydrochloric acid method.

#### 4.1.3. Salt Roasting-Water Leaching Method

The salt roasting-water leaching method is similar to the salt roasting-water leaching method for lepidolite. First, pollucite is mixed with one or several alkali metal salts and then roasted at a high temperature. After that, the roasted ore undergoes a water leaching reaction at a certain temperature. Rubidium in the leaching solution will be converted into rubidium alum salt, and then RbCl can be directly obtained through the extraction process.

The Salt Roasting-Water Leaching Method is one of the common processes for extracting rubidium and cesium resources from pollucite. The process is simple and easy to operate, but the roasting temperature required is 800–1000 °C, making it unsuitable for large-scale industrial production. The rubidium and cesium extraction rate of the Salt Roasting-Water Leaching Method is only about 80–90%, and further separation and extraction of other valuable elements in pollucite are required.

### 4.2. Rubidium Extraction from Potash Feldspar

Potash feldspar (K_2_O·Al_2_O_3_·6SiO_2_) is an aluminosilicate mineral of alkaline earth metals, and the rubidium content in potash feldspar is approximately 3% [67]. Rubidium and cesium resources can be extracted and recovered from rubidium-bearing feldspar using acid, alkali and salt methods. Essentially, whether by the acid method, alkali method or salt method, the process involves reacting potash feldspar with acids, alkalis or salts [68], converting the feldspar into rubidium and cesium salts, which are ultimately extracted in the form of soluble compounds.

#### 4.2.1. Acid Leaching Method

The acid leaching method is used to heat feldspar in a resistance furnace for pre-leaching, followed by further acid leaching of leaching residue to dissolve the feldspar, and Rb and Cs ions in the feldspar are dissolved so as to realize the extraction of rubidium and cesium resources. When hydrochloric acid was used in combination with chloride salt, the extraction effect of Rb was not satisfactory. In contrast, when sulfuric acid was mixed with CaF_2_ as the leaching agent, fluorine could promote the dissolution of feldspar, and the recovery of rubidium showed a significant upward trend with the increase in CaF_2_. However, fluorine is introduced into the reaction process and the remaining solution contains a large amount of acid, which makes subsequent treatment complicated. Therefore, for potassium feldspar, the acid leaching method does not have the prospect of large-scale industrial production, and improvements and innovations in the method should be considered. In recent years, the integration of electrochemical and other processes has shown considerable development prospects. This approach enables the enrichment and efficient extraction of rubidium and cesium by enhancing roasting and leaching processes under mild conditions.

#### 4.2.2. Salt Roasting-Water Leaching Method

The salt roasting method essentially utilizes the sintering method [69] to agglomerate feldspar particles, thereby simplifying the extraction of rubidium. Potassium feldspar is mixed evenly with one or both of sodium chloride and calcium chloride in a certain proportion and then roasted to release rubidium ions from the silicate minerals. The roasted ore is subjected to a water leaching process, the leach solution is desiliconized and then the rubidium and cesium resources are obtained after a solvent extraction process. The salt roasting method features a simple and environmentally friendly process with a high rubidium recovery rate. Meanwhile, coexisting ions exist in the leachate in the form of soluble salts, which can be synchronously recovered during the extraction process, thereby enhancing the additional value of the products.

Given the low concentrations of rubidium and cesium in potassium feldspar, the key technical challenge for acid-based methods and salt roasting methods lies in how to achieve their enrichment and efficient extraction. Rational optimization of leaching processes and the development of green leaching agents are currently the main development directions.

## 5. Potential Resource

With the further development of research, many potential resources containing rubidium and cesium have been gradually discovered. For example, in solid minerals such as kaolin wastes and flotation tailings containing rubidium/cesium [70], and in rubidium/cesium-containing solutions such as geothermal water and radioactive wastewater.

### 5.1. Flotation Tailings

The content of rubidium and cesium in tailings is low, only about 0.15–0.7%, but the large amount of tailings can greatly alleviate the shortage of rubidium and cesium supply. For rubidium and cesium-containing tailings current studies mainly use traditional leaching methods, including the acid method, salt method and roasting method [71,72,73].

The traditional leaching method essentially involves roasting the tailings in a mixture with acid or salt, where rubidium and cesium in the tailings are mainly endowed in the structure of the undamaged mineral phase, which is completely destroyed by the introduction of calcium and fluoride ions during the roasting process, which contributes to the complete destruction of the mineral phase [74]. Rubidium and cesium ions enter the liquid through the water leaching process, and the rubidium and cesium products are obtained by the process of evaporation and crystallization of the leachate and extraction and purification. In total, 80–95% recovery of rubidium and cesium can be achieved by roasting and water leaching. Wang [75] introduced Ca^2+^ in the roasting process, using CaCl_2_ and NaCl-composite salt chloride mixed with tungsten tailings in proportion. After roasting at 950 °C for 2 h, the roasted tailings were mixed with pure water at 1:1.5, the water leaching reaction was carried out for 1 h, and the final rubidium leaching rate was basically stable at 88–89%. Fang [76] studied fluorite flotation tailings containing low-grade rubidium using a mixed acid (H_2_SO_4_ + H_2_SiF_6_) leaching process, where fluorine greatly disrupted the mineral structure, allowing rubidium to be stored in solution as a sulphate. The optimum process conditions were as follows: H_2_SO_4_ in the amount of 60% and H_2_SiF_6_ in the amount of 20% were mixed with the tailings in a mass ratio of 2.5:1, and the water leaching reaction was carried out at 95 °C for 1 h. Microwave irradiation over conventional heating conditions resulted in a rubidium leaching rate of 83.6%.

### 5.2. Kaolin

**Figure 21:**
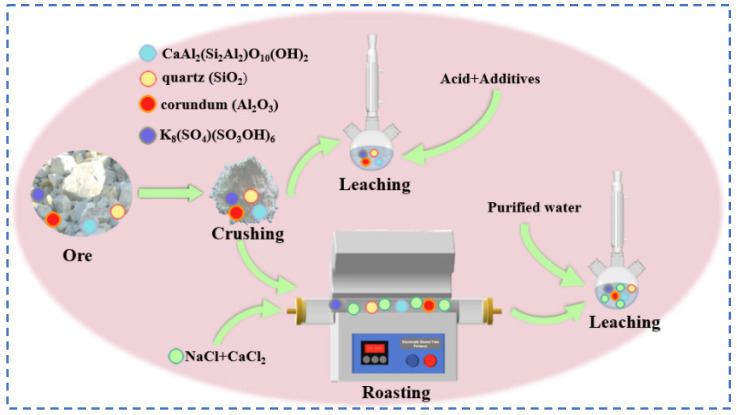
Flow charts for direct acid leaching and water leaching by roasting with chlorinated salts.

The kaolinite crystal chemical formula for the 2SiO_2_·Al_2_O_3_·2H_2_O class of minerals belongs to the 1:1 type layered silicate minerals. The crystals are mainly composed of silica–oxygen tetrahedra and aluminum hydroxide octahedra. The structure is similar to the lithium mica, accompanied by a trace of rubidium resources, and the use of the traditional leaching method can extract it directly from rubidium resources, which has certain research prospects. Zhou [77], for kaolin waste containing 0.208% rubidium, explored the traditional direct acid leaching method and chlorinated salt roasting water leaching method, as shown in Figure 21. The acid leaching method uses hydrochloric acid and sulfuric acid mixed with CaF_2_ for the leaching reaction of kaolin. With the hydrochloric acid leaching method, the rubidium leaching rate is low. With the sulfuric acid leaching method, the rubidium leaching rate is as high as 95.5%, but the subsequent excessive H^+^ will make it difficult to remove Ca^2+^, resulting in a large number of gel-like precipitates. Treatment procedures are complex and costly, not suitable for practical industrial applications. Therefore, focusing on the chloride salt roasting and water leaching process, kaolin was mixed with CaCl_2_:NaCl in the mass ratio of 1:0.3:0.2 at 900 °C for 30 min, and the leaching reaction was carried out between the roasted minerals and pure water in a solid–liquid ratio of 1:2 for 1 h to achieve 90.6% extraction of rubidium. The salt roasting method can recover valuable elements such as rubidium and cesium in kaolin to a great extent, and the process flow is easy to operate.

### 5.3. Geothermal Water

It is widely recognized that geothermal water contains naturally occurring valuable metals and serves as a highly promising alternative source for resource recovery. Influenced by geographical location, the distribution and composition of valuable metals in geothermal water show significant differences (as shown in Table 5). Jiang [78] synthesized a novel adsorbent—potassium iron antimonothiolate (PIATS)—which exhibits high adsorption capacity for cesium. High selectivity and high adsorption capacity can enable the green recovery of cesium resources from geothermal water. When PIATS comes into contact with geothermal water containing coexisting ions, it can complete the efficient and selective adsorption of Cs^+^ in only 3 min. The cesium-loaded PIATS was eluted using 0.50 mol/L NaNO_3_ solution, the rapid desorption could be completed in 10 min, and the concentration of Cs^+^ in the eluent was more than 11 times higher than that in the original solution. The whole process did not involve organic solvents or other toxic reagents. With its high-capacity adsorption, excellent enrichment ability, and stable structural properties, PIATS demonstrates significant potential in the green recovery of cesium resources from geothermal water.

### 5.4. Radioactive Wastewater

For radioactive wastewater [83,84], the extraction of cesium resources mainly involves methods such as adsorption, ion exchange and biosorption. Ion exchange has high selectivity, radiation resistance, and thermal stability, and is prone to removing cesium (Cs^+^) from water. However, this method is limited by its exchange capacity, and the stability of the adsorption reaction is not high [85,86]. Therefore, current research efforts are primarily focused on enhancing the stability of adsorbents and the selectivity of ion exchangers. Currently, studies have been conducted to explore the effect of various ion exchangers such as zeolite, potassium zinc hexacyanoate, silicotitanate, hexacyanoferrate and diammonium titanate on the extraction of cesium. Among them, potassium [87] zinc hexacyanoate showed high cesium adsorption efficiency, excellent results and high adsorption capacity in the pH = 2–10 range, which makes it an excellent prospect. Adsorption remains the primary method for extracting cesium (Cs) from wastewater, and iron cyanide-based materials with excellent ion exchange capabilities hold great potential for exploration in future research.

## 6. Conclusions

As rare metallic elements, rubidium and cesium resources hold irreplaceable strategic application value in traditional industries and high-tech industries due to their unique physical and chemical properties. Rubidium and cesium mainly occur as trace components associated with hard-rock minerals and salt lake brines. In actual industrial production, a complete extraction process system has been formed for rubidium and cesium resources in hard rock minerals such as micas and silicates. Through the combination of acid, alkali and salt media with processes such as roasting, curing or leaching, the efficient extraction of rubidium and cesium resources is realized. For rubidium and cesium resources in salt lake brines and lithium extraction mother liquor, solvent extraction, adsorption, and precipitation methods are commonly employed, enabling the efficient recovery of over 90% of rubidium and cesium.

To alleviate the current shortage in the supply of rubidium and cesium resources, the pressure of resource scarcity can be effectively relieved by reasonably expanding the sources of rubidium and cesium. In potential resources such as flotation tailings and kaolin hard-rock minerals, the salt roasting–water leaching method exhibits excellent extraction performance for rubidium and cesium and in resources such as geothermal water and radioactive wastewater, the adsorption method exhibits excellent performance. The extraction processes for rubidium and cesium in potential resources still need to be further improved. Combining other processes such as microwave-assisted technology with leaching processes to strengthen leaching flow shows certain research prospects. In the field of extracting rubidium and cesium from liquids, the development of low-toxicity and high-efficiency extractants has emerged as a research hotspot while balancing environmental protection and economic costs. Among them, novel phenolic extractants and new composite materials have demonstrated remarkable advantages in extracting rubidium and cesium from low-concentration solutions in complex systems, showcasing enormous research potential.

## Figures and Tables

**Table 1:** Rubidium and cesium properties and uses.

Characterization	Purpose
Ultrafine jump frequencies of electrons in the outer layers of atoms	Atomic clocks, frequency standards, satellite navigation, aerospace measurement and control
Photosensitivity	Night vision equipment, optoelectronic equipment
Easily ionized	Magnetic fluid power generation, ion rockets, fuel cells
Biochemical	Sedatives and hypnotics for the treatment of epilepsy
Absorption	Suction agent for vacuum tubes
Radioactivity	Cs^137^ can be used as a radioactive source to treat cancer
Quantum effect	Quantum computing

**Table 2:** Types and distribution of major deposits.

Deposit Type	Main Minerals	Genesis	Typical Deposits
Pegmatite	Cesium garnet, lithium mica, potassium feldspar, cesium beryl, borofluoropotassium, cesium boron clonorite, cesium manganese stellar feldspar	Crystallization of residual magma of the aerogenic-pegmatitic phase	Tanco deposit in Canada, Bikita deposit in Zimbabwe, Sinclair deposit in Australia.
Granite type	Potassium feldspar, skarn, (iron) lithium mica, black mica, white mica and so on	Residual magma crystallization, hydrothermal crystallization or accounting	Inner Mongolia Lime Kiln, Zhaojinggou, Hunan Zhengchong, Guangdong Tiantang Mountain, Gansu Guobao Mountain (China)
Salt Lake	Halite, potash, carnallite.	Evaporation deposition of concentrated brines	Salton Sea Salt Lake Brine, Qaidam Basin, U.S.A.
Brine and hot spring type	Silica waffle, granular opal, colloidal opal, hydromica, chalcedony square quartz.	Concentration or deposition	Tibet Hitching Post, Tibet Gulu (China)

**Table 3:** Major minerals and rubidium and cesium content.

Mineral Name	Chemical Formula	Content of Rb_2_O (%)	Content of Cs_2_O (%)
Cesium garnet	Cs(A1Si_2_O_6_) nH_2_O	0.3–1.4	23.5–36.5
potassium feldspar	K_2_O·A1_2_O_3_·6SiO_2_	3	-
Lithium Mica	K(Li,A1)_3_(Si,A1)O_10_(OH,F)_2_	3.75	0.4–1

**Table 4:** Precipitants and applications.

Precipitant	Precipitation Form	Characteristics
Silicomolybdenum (tungsten) acid	Rb_4_H_4_[Si(Mo_2_O_7_)_6_], Cs_8_[Si(Mo_2_O_7_)_6_]	High recovery of Rb, Cs but precipitated compounds are unstable and prone to decomposition
Potassium iodobismuthate	M_3_B_2_I_9_	Recovery of Rb, Cs product purity of nearly 100 per cent, the process is complex and requires secondary purification
Chloroplatinic acid	M_2_(PCl_6_)	Rb, Cs recovery is high and expensive
Stannum tetrachloride	M_2_SnCl_6_	Simple process, but time-consuming and costly
Antimony trichloride	3MC1·2SbCl_3_	The process is easy to operate but the cost of chemicals is high
Iodine chloride	MICl_2_	Recovered Rb, Cs products are nearly 100 per cent pure, but the crystallization process needs to be repeated
Aluminum sulfate	MAI(SO_4_)_2_·12H_2_O	No pollution to the environment, but the process is complex and difficult to operate

**Table 5:** Contents of alkali metals such as rubidium and cesium in geothermal waters of different regions [43].

Geothermal Brine	Ion/Metal Concentration (mg/L)						Ref.
	Rb	Cs	Li	Na	Mg	K	
Salton Sea (USA)	170	20	194	53,000	33	16,700	[79]
Brawley (USA)	67	19	219	47,600	114	126,000	[80]
Wairakei (New Zealand)	2.90	2.50	13.2	1250	0.04	210	[81]
Salak (Indonesia)	5	4.50	17	5000	0.10	990	[82]
Cerro Prieto (Mexico)	11	39	27	8300	0.50	2210	[83]
Hvergerdi (Iceland)	0.04	<0.02	0.30	212	-	27	[84]
Mote Amiata (Italy)	2.10	0.70	21.90	1977	<0.50	558	[85]

## Data Availability

No new data were created or analyzed in this study. Data sharing is not applicable to this article.
